# Prevalence and Molecular Characterization of Parasitic Lice in Tibetan Yaks, Pigs and Sheep

**DOI:** 10.3390/life15030444

**Published:** 2025-03-12

**Authors:** Wanmei Luo, Xialing Zhao, Dengyun Wang, Bin Shi, Shah Nawaz, Qingxia Wu, Wenqiang Tang

**Affiliations:** 1 College of Animal Science, Xizang Agriculture and Animal Husbandry University, Nyingchi 860000, China; luowm168@163.com; 2 Tibet Academy of Agriculture and Animal Husbandry Sciences, Lhasa 850009, China; xznmzxl@126.com (X.Z.); 1898902737@163.com (B.S.); 3 Nierong County Agriculture and Animal Husbandry Science and Technology Service Station, Naqu 853500, China; xznrxsfz@163.com; 4 College of Veterinary Medicine, Huazhong Agricultural University, Wuhan 430079, China; malikshahnawaz786@gmail.com

**Keywords:** ectoparasites, lice infestation, phylogenetic analysis, *rrnS* gene, *Haematopinus apri*

## Abstract

The infestation of ectoparasites poses a severe problem in animal breeding, severely affecting animal health and causing substantial economic losses. However, limited information is available regarding lice infestations in plateau livestock. To address this, we collected and examined lice samples from Tibetan yaks, pigs and sheep, amplifying the *rrnS* and *cox1* genes for evolutionary analysis. The results revealed that the prevalence of lice was 51.3% (95% CI: 44.0–58.6%) in yaks, Tibetan sheep and Tibetan pigs, with regional prevalence ranging from 7.7% to 67.5%. Morphometric analysis showed that female lice were bigger than male lice. In Tibetan pigs, females exhibited a prominent longer body length (*p* < 0.05), belly length (*p* < 0.01) and body length to body width ratio (*p* < 0.05). In yaks, females had longer body length (*p* < 0.01) and body width (*p* < 0.05). For Tibetan sheep, female lice had larger body length (*p* < 0.001), body width (*p* < 0.001), head length (*p* < 0.05) and belly length (*p* < 0.05). Additionally, molecular identification revealed that lice infesting wild and domestic yaks were *Linognathus vituli* with 96.59–98.78% (wild yaks) and 96.33–98.71% (yaks) similarity to the OL677823.1 isolate. Lice infesting Tibetan sheep were identified as *Linognathus africanus*, showing 99.02–99.76% similarity to the OP948898.1 isolate. Lice from Tibetan pigs were identified as *Haematopinus apri*, with 96.38–98.31% similarity to the ON000922.1 isolate. Moreover, *cox1* gene analysis of lice from Tibetan pigs showed 96.97–97.72% similarity to the KC814616.1 isolate. It is concluded that these findings could provide valuable insights into the prevention and control of lice-related diseases in plateau animals, enhancing animal health and mitigating economic losses.

## 1. Introduction

The yak (*Bos grunniens*) is a long-haired, large ruminant breed [1], which is mainly distributed on the cold plateau regions of the Himalayas in South Asia with an average altitude of more than 3 km [2]. Globally, more than 16 million yaks inhabit the Qinghai–Tibet plateau in China [3]. In addition, yaks are crucial for both food and economic purposes, providing nutritious meat, high-protein milk, medicinally valuable fur and bones and dung used as fuel by local communities [4].

The Tibetan sheep (*Ovis aries*) is a representative indigenous herbivore inhabiting plateau regions at 3000 m above sea level (MASL) with over 23 million in China [5]. Analogous to yaks, Tibetan sheep have developed good physiological and genetic adaptations to overcome the plateau’s unfavorable environmental conditions, including extreme cold and hypoxia, through prolonged acclimatization. The acclimatization is achieved by several physiological adaptations, including elevated hemoglobin content and elevated mitochondrial content, and elevated oxygen-use efficiency in low oxygen states [6]. Moreover, Tibetan sheep provide a wide range of products, including high-quality meat, wool, fur and milk. These substantial contributions have played a vital role in supporting human civilization in the plateau regions, particularly among Tibetan communities [7]. The major types of sheep breeds found in the Tibetan Plateau region of China include Tibetan Sheep, Pianma Sheep, Ganjia Tibetan Sheep and Oula Sheep. In the present study, sampling was performed randomly.

The small Tibetan pig (*Sus scrofa domesticus*) is a unique local Tibet breed found in the high-altitude regions (3000 MASL) of the southeast Qinghai–Xizang plateau regions [3,8]. Through long-term domestication in harsh conditions characterized by hypoxia, extreme cold, high ultraviolet radiations and limited food resources, this economically significant, free-ranging species has developed exceptional resilience to disease and environmental stress [2]. These pigs produce not only lean meat with high yield but also high-quality, nutrient-rich products abundant in protein and essential amino acids [2]. However, the occurrence of infectious diseases in these animals poses a significant risk, leading to reduced productivity and substantial economic losses to animal husbandry [8,9,10].

Compulsory blood-sucking ectoparasites are widely recognized as a serious health threat to livestock, causing anemia and great economic losses by impairing rumination, reducing weight gain and lowering the quality and quantity of meat and milk production [11,12]. In Brazil, the estimated annual losses in the cattle industry due to ectoparasites was USD 6.86 billion [13], while Australia’s sheep industry suffered annual losses of USD 81 million [14]. Beyond their direct effects, many ectoparasites serve as vectors for blood-borne pathogens, promoting the spread of diseases among animals [15]. Among them, *Linognathus vituli*, a common ectoparasite of cattle, has a global prevalence ranging from 5% to 96% [16]. In addition to causing direct harm to ruminants and bringing economic losses to the cattle industry [17], this parasite is a crucial adjective vector of louse-borne pathogens such as *Anaplasma marginale*, *Theileria orientalis*, *Rickettsia* spp. and *Coxiella burnetti* [11,17,18]. Similarly, *L. africanus* is a major external parasite of sheep and goats [19], leading to significant economic losses for farmers and the tanning industry [20]. Alarmingly, zoonotic pathogens like *Rickettsia* sp. were detected in *L. africanus*, highlighting its public health risk [21]. Furthermore, the blood-sucking ectoparasite *Haematopinus apri*, commonly found in wild pigs, is a critical vector for pathogens such as the African swine fever virus, classical swine fever virus, *Anaplasma* spp. and the swinepox virus, posing a severe threat to livestock health and productivity [22].

Although lice are recognized as seriously harmful vermin, limited knowledge exists about *L. vituli*, *L. africanus* and *H. apri* infections in plateau animals (Family: *Pedicinidae*, Genus, *Linognathus* and *Haematopinus*). Therefore, the current study was conducted to investigate the prevalence and molecular characterization of these parasitic lice in yaks, Tibetan pigs and Tibetan sheep for the first time, assessing their impact on high-altitude yaks, pigs and sheep.

## 2. Materials and Methods

### 2.1. Sample Collection

**Figure 1:**
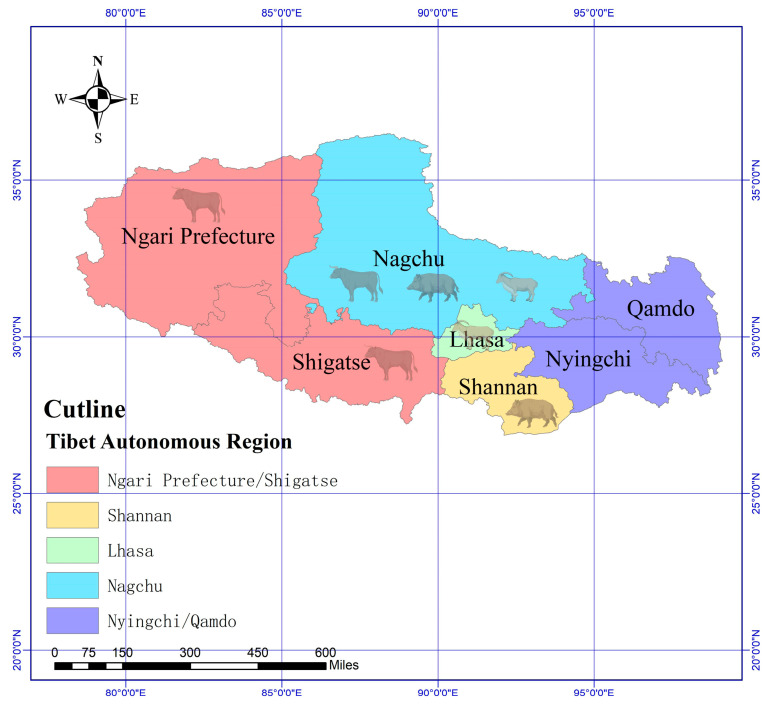
Mapping the origins—geographical locations of sample collection.

Lice samples were collected from domestic yaks (n = 77), a wild yak (n = 1), Tibetan pigs (n = 20) and Tibetan sheep (n = 91) on the Tibetan plateau during 2022–2023. Samples were obtained by scraping the back skin of animals with sterile cotton swabs, while larger lice were directly picked using tweezers. All collected samples were fixed in 75% ethanol and stored at −20 °C for subsequent analysis [8]. The primers sequence is provided in Table 1 and the geographical locations of sampling are shown in Figure 1.

### 2.2. Lice Examination and Morphological Identification

All swab samples were examined using ZEN (blue edition) microscopy software (Version: 2.5) (ZEISS, Germany), and morphological identifications were performed following the methods described in previous studies [23,24]. A comprehensive measurement using morphometric analysis to obtain principal anatomical parameters of the specimens was undertaken including body length (BL/mm), body width (BW/mm), head length (HL/mm), belly length (BeL/mm), chest length (CL/mm) and breadth of breastplate (BBP/mm), and these specimens were carefully measured under the microscope to obtain accurate and reliable data. Additionally, the body length to body width ratio (LW) was calculated to assist in species characterization.

### 2.3. DNA Extraction and rrnS and cox1 Genes Amplification

Genomic DNA of yaks, Tibetan sheep and Tibetan pig lice were purified using the PureLink™ Genomic DNA Purification Kit (Invitrogen, Carlsbad, CA, USA) following the manufacturers’ strict protocol. The purified DNA thus acquired was then used to serve as templates for amplifying two candidate genes: mitochondrial ribosomal RNA small subunit (*rrnS*) and cytochrome c oxidase subunit 1 (*cox1*). The primer information is shown in Table 1. For PCR amplification, 50 μL reactions were prepared, including 25 μL 2 × Hieff^®^ PCR Master Mix (Yeasen Biotechnology Co., Ltd., Shanghai, China), 2 μL DNA, 1 μL forward primer (10 μM), 1 μL reverse primer (10 μM) and 21 μL nuclease-free water. The PCR conditions were as follows: initial denaturation at 95 °C for 2 min, followed by 35 cycles of 95 °C for 30 s, annealing at 64 °C/*cox1* (62 °C/*rrnS*) for 45 s, 72 °C for 30 s and a final extension at 72 °C for 10 min. PCR products were analyzed using 1.5% agarose gel electrophoresis to verify amplification.

### 2.4. Sequencing and Phylogenetic Analyses

All the PCR positive bands were purified using the MolPure^®^ Gel Extraction Kit (Molpure, Laguna Hills, CA, USA) and subsequently sent for commercial sequencing at Sangon Biotech (Shanghai, China). The sequences obtained then aligned with reference genes, including *cox1* and *rrnS*, using NCBI BLASTn and MEGA (Version: 11.0). The reference genes for *rrnS* included sequences from *Linognathus vituli* (HM241899.1, L677823.1), *Linognathus africanus* (OP948898.1, HM171397.1), *Linognathus spicatus* (HM171399.1), *Haematopinus apri* (ON000922.1, ON000918.1) and *Pedicinus badii* (FJ267403.1). For *cox1*, the reference sequences were from *Haematopinus apri* (KC814616.1, ON000917.1), *Linognathus vituli* (HM241900.1), *Linognathus africanus* (OP948899.1) and *Pedicinus badii* (MT721727.1). Phylogenetic analysis of the *cox1* and *rrnS* genes was carried out via MEGA (11.0) to assess the phylogenetic relationships between lice derived from plateau animals and other available lice isolates [25]. A phylogenetic tree was constructed using the maximum likelihood method, and the firmness of branches was evaluated by bootstrapping with 1000 times recalculation [26].

### 2.5. Statistical Analysis

The infestation rates of lice in different animals and regions, as well as body indexes of different lice species, were statistically analyzed using the chi-square test in SPSS (18.0) and the multiple unpaired *t*-test in GraphPad Prism (9.0). Confidence intervals were calculated using the Clopper–Pearson method. A significant difference was considered when *p* < 0.05.

## 3. Results

### 3.1. The Prevalence of Lice in Yaks, Tibetan Sheep and Tibetan Pigs

**Figure 2:**
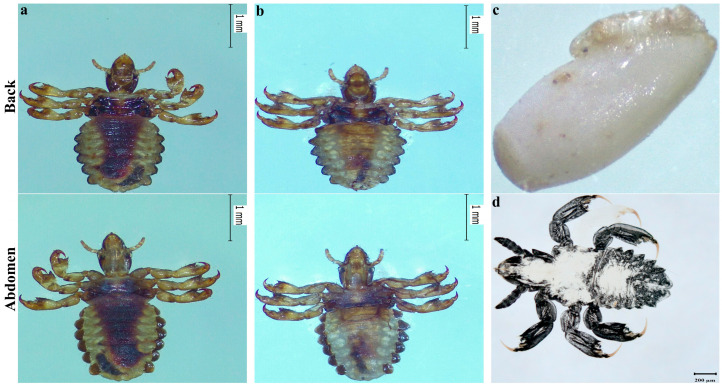
Morphological presentation of lice in Tibetan pigs: (**a**) female, (**b**) male, (**c**) ovum, (**d**) nymph.

**Figure 3:**
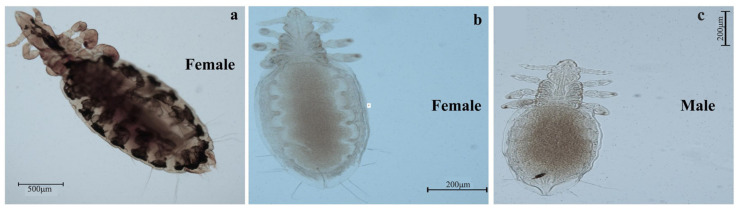
Morphological presentation of lice in yaks (**a**) and Tibetan sheep (**b**,**c**).

**Figure 4:**
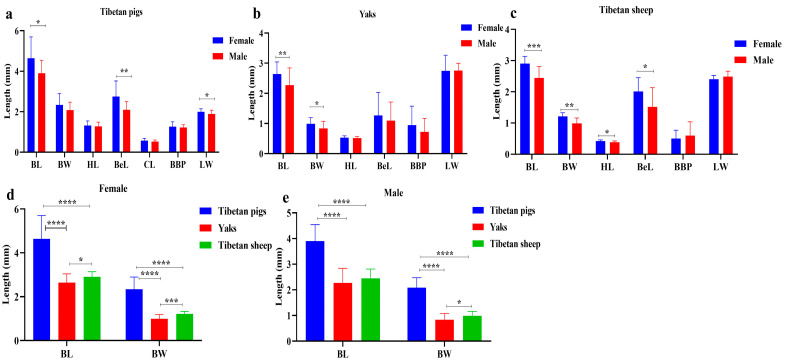
Comparative analysis of body index of lice from different animals: (**a**) lice in Tibetan pigs, (**b**) lice in yaks, (**c**) lice in Tibetan sheep, (**d**) comparing female lice from different animals (Tibetan pigs, Yaks, and Tibetan sheep), (**e**) comparing male lice from different animals (Tibetan pigs, Yaks, and Tibetan sheep). * *p* < 0.05, ** *p* < 0.01, *** *p* < 0.001, **** *p* < 0.0001.

A total of 97 animals were examined, with an overall lice prevalence of 51.3% (95% CI: 44.0–58.6%). The prevalence varied across regions, ranging from 7.7% to 67.5%, showing a significant difference among different areas (*p* < 0.01). The prevalence of lice also differed significantly among animal species, ranging from 18.2% to 100.0% across different breeds (*p* < 0.01) (Table 2). Moreover, Figure 2a–c and Figure 3a–c show the morphological presentation of lice in yaks, Tibetan sheep, and pigs. Furthermore, Figure 4 shows the comparative analysis of the body index of lice from Tibetan pigs, yaks, and Tibetan sheep (Figure 4a–c), and also compares the female and male lice from different animals (Tibetan pigs, Yaks, and Tibetan sheep), (Figure 4d,e).

### 3.2. Molecular Characterization of Lice in Yaks, Tibetan Sheep and Tibetan Pigs

**Figure 5:**
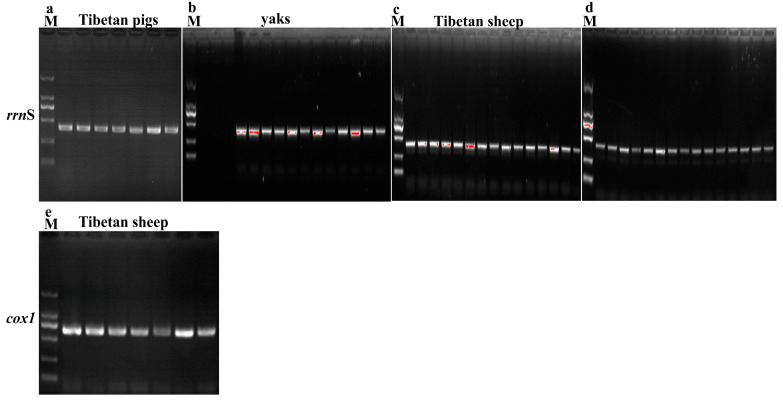
PCR amplification results of the *rrnS* and *cox1* genes of lice from yaks, Tibetan sheep and Tibetan pigs on 1.5% agarose gel: (**a**) *rrnS* gene of lice from Tibetan pigs, (**b**) *rrnS* gene of lice from yaks, (**c**) *rrnS* gene of lice from Tibetan sheep, (**d**) *rrnS* gene of lice from wild yak, (**e**) *cox1* gene of lice from Tibetan sheep. Marker: 2000, 1000, 750, 500, 250, 100.

The mitochondrial *rrnS* gene of lice from Tibetan pigs, yaks and Tibetan sheep, as well as the *cox1* gene of lice from Tibetan pigs, were successfully amplified (Figure 5). The PCR products corresponding to the *cox1* gene were subsequently sequenced and submitted to the NCBI database with accession numbers of PQ615368-PQ615374 (Tibetan pigs from Nagchu) and PQ615375-PQ615378 (Tibetan pigs from Shannan). The *rrnS* sequences were assigned the following accession numbers: PQ622861-PQ622867 (Tibetan pigs from Nagchu), PQ622868-PQ622882 (Tibetan pigs from Shannan), PQ622839-PQ622845 (Tibetan sheep from Lhasa), PQ622846-PQ622860 (Tibetan sheep from Nagchu), PQ622800-PQ622814 (wild yaks from Ngari), PQ622815-PQ622829 (yaks from Nagchu) and PQ622830-PQ622838 (yaks from Ngari).

BLAST (version 2.15.0) analysis of these sequences with the NCBI database (https://blast.ncbi.nlm.nih.gov/Blast.cgi?PROGRAM=blastn&PAGE_TYPE=BlastSearch&LINK_LOC=blasthome. Accessed on 5 November 2023) revealed that lice from wild yaks and domestic yaks were identified as *L. vituli* with 96.59–98.78% (wild yaks) and 96.33–98.71% (domestic yaks) similarity to OL677823.1 isolated from cattle in Changsha, China, via the *rrnS* gene. The lice infected in Tibetan sheep were identified as *L. africanus* with 99.02–99.76% similarity to OP948898.1 isolated from *Capra hircus* in Pakistan via the *rrnS* gene. Lice infected in Tibetan pigs were identified as *H. apri* with 96.38–98.31% similarity to ON000922.1 isolated from wild boar in China via the *rrnS* gene. The *cox1* gene from lice in Tibetan pigs was 96.97–97.72% similar to KC814616.1 isolated from *Capra hircus* in Pakistan.

**Figure 6:**
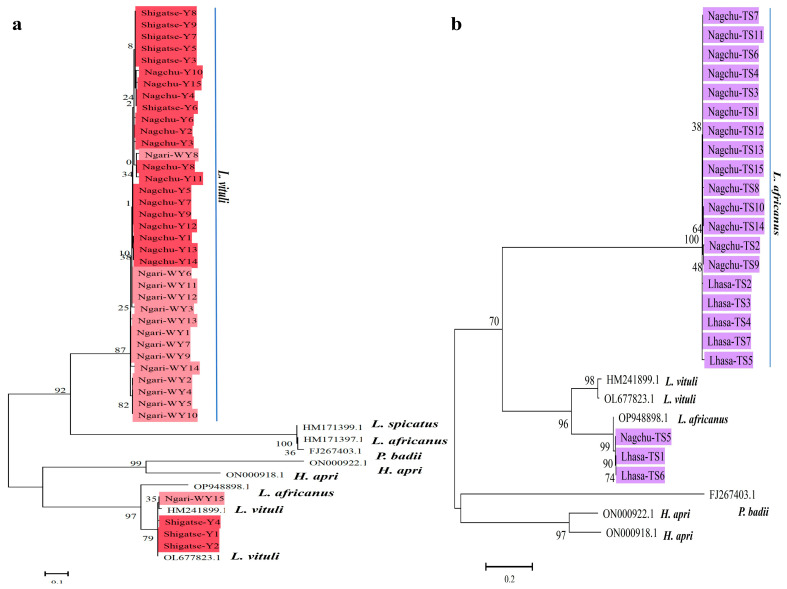
Phylogenetic analysis of the current lice mt genomes of lice, focusing on the *rrnS* sequences using the maximum likelihood method. The resultant phylogenetic trees are presented with bootstrap values: (**a**) phylogenetic tree of lice from yaks based on the *rrnS* gene, (**b**) phylogenetic tree of lice from Tibetan sheep based on the *rrnS* gene.

**Figure 7:**
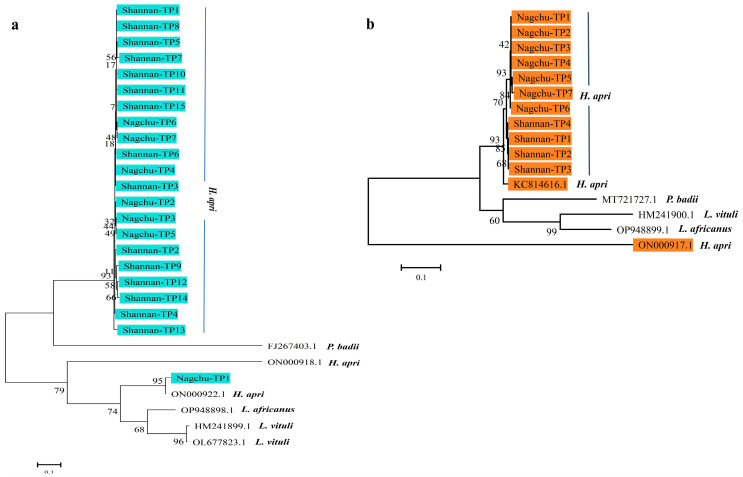
Phylogenetic analysis of the current lice mt genomes of the *rrnS* and *cox1* sequences with references through the maximum likelihood method. The numbers at clades present bootstrap values: (**a**) phylogenetic tree of lice from Tibetan pigs based on the *rrnS* gene, (**b**) phylogenetic tree of lice from Tibetan pigs based on the *cox1* gene.

Phylogenetic analysis of the *rrnS* gene showed that the *L. vituli* isolate from wild yaks (Ngari-WY15) and three isolates from yaks (*Shigatsw-Y1, Shigatsw-Y2, Shigatsw-Y4*) clustered with HM241899.1, while other isolates formed an independent branch (Figure 6a). Similarly, *L. africanus* isolates (*Lhasa-TS1, Nagchu-TS5, Lhasa-TS6*) from Tibetan sheep were located in the same cluster with OP948898.1, while other isolates formed a distinct cluster (Figure 6b). The *H. apri* isolate from Tibetan pigs (*Nagchu-TP1*) clustered with ON000922.1, while other isolates were placed in a separate cluster (Figure 7a). Additionally, all *H. apri* isolates from Tibetan pigs clustered with KC814616.1 based on the *cox1* gene (Figure 7b).

## 4. Discussion

Food animals have a close relationship with the sustainable development of the economy and human civilization, and safeguarding animal health is meaningful and vitally important [27]. Despite their considerable impact as harmful ectoparasites, lice remained somewhat under-studied, particularly for their counterparts on agricultural livestock [28]. To the best of our knowledge, this current study is the initial probing of lice of veterinary importance on plateau animals regarding their molecular profiles in China.

The overall prevalence of lice in Tibetan livestock was determined to be 51.3%, comparable to the values reported in Irish cattle [29]. Caution must be exercised in directly comparing studies due to inherent variations in host species, breeds, age structure, management systems and geographic locations such as goats (97.4%) and sheep (100%) in Indonesia. For example, the prevalence of *L. vituli* in yaks, at 18.2%, was comparable to 19.3% in Ethiopian cattle [30,31]. However, differences in host physiology and environmental conditions may affect infestation patterns. Similarly, the prevalence of *L. africanus* in Tibetan sheep, at 93.5%, was considerably higher than that in goats of Rampur, at 11.2% [32], and in sheep (11.5%) and goats (27.9%) in Ethiopia [20]. The observed disparity may be due to variations in climatic conditions, ectoparasite control measures and differences in host susceptibility.

Comprehensive research has been conducted on *H. suis* in pigs [27,33]; however, information about *H. apri* in animals is limited. The prevalence of *H. apri* in Tibetan pigs was 50%, which was near *H. suis* in pigs (63.6%) in Rwanda [27], but higher than *H. suis* prevalence in pigs (15.3%) in Argentina [33]. The prevalence of lice varied significantly in different regions and breeds (Table 2) [31,34]. Our findings, along with previous studies, suggest that the occurrence of infestations in animals is determined by a range of factors, including both environmental and host-related factors. Specifically, free-grazing and direct contact with neighboring animals contribute most toward raising infestation intensity, through increased opportunity for parasite transmission. In addition, infestation trends follow a seasonal distribution, with increased infestations in winter in sheep and in goats in warmer environments. Herd size, geographical location and breed susceptibility have been determined to contribute toward infestations, with larger herds and native breed types having a relation with stocking density and genetic susceptibility toward infestations [1,2,3].

Previous studies have reported species such as *H. eurysternus*, *H. quadripertusus*, *H. tuberculatus*, *L. vituli* and *Solenopotes capillatus* in cattle [12,35]. However, all 39 isolates from wild and domestic yaks in our study were identified as *L. vituli*, which could be attributed to the fact that *L. vituli* is one of the most commonly reported lice species in cattle [36]. The lice from yaks exhibited high similarity (96.33–98.71%) to *L. vituli* (OL677823.1) isolated from adult cattle in Changsha, China, via blast analysis. This current study implies a potential genetic affinity between *L. vituli* from plateau yaks and the cattle isolate. However, in the phylogenetic tree based on the *rrnS* gene (Figure 6a), *L. vituli* isolates clustered into two distinct groups, possibly due to a few base differences among the isolates and the relatively short length of the *rrnS* gene.

Unlike previous studies, which primarily focused on *H. suis* in pigs [33,37], all 22 isolates from infected Tibetan pigs in our study were identified as *H. apri* with high similarity (96.38–98.31%) to the *H. apri* isolate (ON000922.1) from wild boar via the *rrnS* gene. These findings were further confirmed by analysis of the *cox1* gene (Figure 7). *H. apri* is a louse species typically found in wild boars, and it shares both morphological and phylogenetic similarities with *H. suis* [38]. The occurrence of such a species of lice, endemic in feral pigs and wild boars naturally, can best be explained in terms of several ecologic and epidemiologic factors. One such reason is sympatric coexistence and reduced contact between feral and domestic pigs in a shared habitat with spillovers of parasites. Intermediate hosts such as birds and rodents can transmit infection through mechanically transporting lice and eggs between feral and domestic animals. In sheep and goats, common lice species include *Bovicola caprae*, *B. limbata*, *B. crassipest*, *B. ovis*, *L. africanus* and *L. stenopsis* [14,19]. In particular, *B. ovis* is frequently reported in sheep [39]. In contrast, all 22 isolates from Tibetan sheep in our study were identified as *L. africanus*, with high similarity (99.02–99.76%) to isolate (OP948898.1) from *Capra hircus* in Pakistan via the *rrnS* gene. *L. africanus* is a goat louse [40], which was reported in *Capra hircus* [28] and goats [19], and seldom detected in sheep. Our findings suggest that *L. africanus* may also infest sheep. The close cohabitation of Tibetan sheep and goats in shared grazing pastures, which could facilitate cross-species transmission, could be one of the possible reasons of the unusual presence of *L. africanus*. It is known that these plateau animals are social livestock that coexist with many wild animal species [1]. Lice are an important vector-borne reservoir for micro-pathogens [41]; hence, those infested animals may transmit lice and infectious microbes to other animals, particularly during warmer seasons. Zoonotic pathogens such as *Rickettsia* spp. and *Bartonella* spp. transmitted by lice may also pose a health threat to herders in plateau regions [28].

The small sample size of the wild yaks (n = 1) and Tibetan pigs (n = 20) puts limitations on the study’s validity. Sampling from the wild yaks was extremely limited due to their evasive behavior, harsh high-altitude habitat and their conservation status. Nonetheless, molecular characterization provides a useful baseline for future epidemiological investigations. An increase in the intensity of surveillance activities with larger sample sizes and the utilization of non-invasive genetic sampling techniques will be necessary to enhance our understanding of the dynamics of infestation by lice among populations of wild yaks.

## 5. Conclusions

In conclusion, we reported a high prevalence of lice infestation in yaks (18.2%), Tibetan sheep (93.5%) and Tibetan pigs (50.0%) on the Tibetan plateau. Through molecular analysis, lice were identified as *L. vituli*, *L. africanus* and *H. apri*, respectively, with a high genetic similarity to reference isolates. Notably, we confirmed for the first time that *L. africanus*, previously considered a goat louse, can also infest Tibetan sheep in the plateau environments. These findings may make a valuable contribution to the disease prevention and control of lice-related disease on plateau animals.

## Figures and Tables

**Table 1:** Primer information of lice used in the current study.

Target Gene	Name of Primer	Sequence (5′ to 3′)	Size of Target Band (bp)
*cox1*	HACX1F	GCATATAATTATGGAGGAGAGAGGAA	657
	HACX1R	ATGACCAAAAAACCAGAATAGATGCTGG	
*rrnS*	HA12SF	GGTCCATGAAAACTAATAACATATGGCGGT	452
	HA12R	CATTGTATATAGTAGGGTATCTAATCCTAG	

**Table 2:** The prevalence of lice in yak, Tibetan sheep and Tibetan pigs.

Variable	Category	No. Examined	No. Positive	% (95% CI)
Region ^a^	Lhasa	26	2	7.7% (0.9–25.1%)
	Ngari	20	3	15.0% (3.2–37.9%)
	Shigatse	9	4	44.4% (13.7–78.8%)
	Shannan	11	5	45.5% (16.7–76.6%)
	Nagchu	123	83	67.5% (58.4–75.6%)
Breed ^b^	Domestic yak	77	14	18.2% (10.3–28.6%)
	Tibetan pig	20	10	50.0% (27.2–72.8%)
	Tibetan sheep	91	72	93.5% (85.5–97.9%)
	Wild yak	1	1	100.0% (2.5–100.0%)
Total		189	97	51.3% (44.0–58.6%)

^a^ Significant difference was detected of the prevalence of lice in animals in different regions (*p* < 0.001, χ^2^ = 43.548). ^b^ Significant difference was detected of the prevalence of lice in animals in different regions (*p* < 0.001, χ^2^ = 62.962).

## Data Availability

The *rrnS* and *cox1* sequences of lice were deposited in GenBank with accession number: PQ622800-PQ622838, PQ622839-PQ622860 and PQ622861-622882.
